# Emerging Roles of Autophagy and Inflammasome in Ehrlichiosis

**DOI:** 10.3389/fimmu.2019.01011

**Published:** 2019-05-08

**Authors:** Tyler R. Tominello, Edson R. A. Oliveira, Shah S. Hussain, Amr Elfert, Jakob Wells, Brandon Golden, Nahed Ismail

**Affiliations:** ^1^Department of Pathology, School of Medicine, University of Pittsburgh, Pittsburgh, PA, United States; ^2^Department of Pathology, College of Medicine, University of Illinois at Chicago, Chicago, IL, United States

**Keywords:** ehrlichiosis, inflammasome, autophagy, pathogenesis, innate immunity, mechanism

## Abstract

Human monocytic ehrlichiosis (HME) is a potentially life-threatening tick-borne rickettsial disease (TBRD) caused by the obligate intracellular Gram-negative bacteria, *Ehrlichia*. Fatal HME presents with acute ailments of sepsis and toxic shock-like symptoms that can evolve to multi-organ failure and death. Early clinical and laboratory diagnosis of HME are problematic due to non-specific flu-like symptoms and limitations in the current diagnostic testing. Several studies in murine models showed that cell-mediated immunity acts as a “double-edged sword” in fatal ehrlichiosis. Protective components are mainly formed by CD4 Th1 and NKT cells, in contrast to deleterious effects originated from neutrophils and TNF-α-producing CD8 T cells. Recent research has highlighted the central role of the inflammasome and autophagy as part of innate immune responses also leading to protective or pathogenic scenarios. Recognition of pathogen-associated molecular patterns (PAMPS) or damage-associated molecular patterns (DAMPS) triggers the assembly of the inflammasome complex that leads to multiple outcomes. Recognition of PAMPs or DAMPs by such complexes can result in activation of caspase-1 and -11, secretion of the pro-inflammatory cytokines IL-1β and IL-18 culminating into dysregulated inflammation, and inflammatory cell death known as pyroptosis. The precise functions of inflammasomes and autophagy remain unexplored in infections with obligate intracellular rickettsial pathogens, such as *Ehrlichia*. In this review, we discuss the intracellular innate immune surveillance in ehrlichiosis involving the regulation of inflammasome and autophagy, and how this response influences the innate and adaptive immune responses against *Ehrlichia*. Understanding such mechanisms would pave the way in research for novel diagnostic, preventative and therapeutic approaches against *Ehrlichia* and other rickettsial diseases.

## The Agents of Ehrlichiosis

Ehrlichiosis is caused by *Ehrlichia*; an obligate intracellular Gram-negative bacteria that belongs to Anaplasmataceae family of the order Rickettsiales. Other genera within the family Anaplasmataceae are: *Anaplasma, Neorickettsia*, and *Wolbachia* (1). Ehrlichiae are maintained in natural cycles throughout persistently infected vertebrate hosts and tick vectors (2–4).

## Clinical Presentations

###  Human Monocytic Ehrlichiosis (HME)

*Ehrlichia* infection is recognized as an important public health threat in tick-endemic areas including the south and south-central regions of the US, although a global threat also exists due to the prevalence of HME worldwide. *Ehrlichia chaffeensis* is the foremost etiologic agent of HME, however, other *Ehrlichia* species, including *Ehrlichia canis, Ehrlichia muris* and *Ehrlichia muris*-like agent (EMLA) are also isolated from patients with HME (5). The vector for *E. chaffeensis* is *Amblyomma americanum*, which is also vector for *Rickettsia* species such as *R. amblyommii* and *R. parkeri* (4).

Human monocytic ehrlichiosis presents as an acute febrile illness associated with fever, malaise, myalgia, and headache. Clinical manifestations of skin and gastrointestinal tract involvement such as rash, nausea, vomiting, diarrhea, and regional lymphadenopathy occurs in 20–40% of patients (4). Meningoencephalitis and pneumonia occur in 20% of patients who present with stiff neck, confusion, cough, and dyspnea. Life-threatening complications such as renal failure, adult respiratory distress syndrome, meningoencephalitis, multi-system organ failure, and toxic shock occur in a substantial portion of the patients who are hospitalized. HME is often undiagnosed or misdiagnosed as a result of non-specific clinical manifestations and lack of specific and sensitive diagnostic tests. Characteristic laboratory findings in HME patients are thrombocytopenia, leukopenia, neutropenia, and increased levels of hepatic transaminases (4, 6, 7). Diagnostic tests such as peripheral blood smear, *in-vitro* culture, PCR and serological testing are currently used to identify HME. However, each of these tests has potential limitations with suboptimal sensitivity or specificity at early stages of infection. Antibiotic treatment with doxycycline (drug of choice) is effective only if given early in infection. Failure to treat immunocompetent patients with doxycycline at the early stages of infection or in infected-immunocompromised individuals often results in serious and progressive disease that mimics septic or toxic shock-like syndrome and multi-organ failure with a case fatality rate of 3%. The clinical, diagnostic and therapeutic challenges in the management of patients with ehrlichiosis account for a high rate of hospitalization (40–63%) (4, 6). Thus, there is a critical need in creating new options for effective countermeasures (e.g., diagnostics, preventive and therapeutic measures) to control these pathogens. Understanding the immunopathogenesis of HME will enable us to develop new avenues for sensitive and specific diagnostic testing during early infection and immunotherapies for later disease management.

###  Canine Ehrlichiosis

*Ehrlichia canis* is the major cause of canine ehrlichiosis, although other human *Ehrlichia* species such as *E. chaffeensis* and *E. ewingii* can also infect dogs. *E. ewingii* is transmitted by the lone star tick, *A. americanum*, while *E. canis* is transmitted by the brown dog tick, *Rhipicephalus sanguineus*. The bacteria are maintained through the tick life stages by transstadial, but not by transovarial, transmission (8, 9). Similar to *E. chaffeensis*, the natural host for *E. canis* is the white-tailed deer, although chronically infected dogs are also considered as reservoirs (10).

*E. canis* and *E. chaffeensis* primarily infect monocytes, thus causing canine monocytic ehrlichiosis, while *E. ewingii* infect granulocytes causing canine granulocytic ehrlichiosis. Dogs with acute canine ehrlichiosis may present with multi-system disease including lymphadenopathy, splenomegaly, ocular signs such as uveitis, retinitis, retinal hemorrhage or retinal detachment (11). Similar to HME, meningoencephalitis or cerebral hemorrhage may occur in 20% of infected dogs and present with stupor, ataxia, central or peripheral vestibular dysfunction, cerebellar dysfunction, convulsion, and tremors. Hematologic and immunologic abnormalities are commonly marked by the presence of petechiae, dermal ecchymosis, and autoimmunity including generation of anti-platelet antibodies that may account for thrombocytopenia, leukopenia, anemia, and hemolysis (10). Laboratory findings in dogs with either subacute or chronic monocytic or granulocytic ehrlichiosis include high serum levels of alkaline phosphatase and/or liver transaminases, hypocalcemia, hypokalemia, hyperglobulinemia, and seroconversion after 7–14 days post-infection (10, 11). Unlike HME, *Ehrlichia* infection in dogs can be self-limited even without antibiotic treatment but can cause persistent/chronic infection. Chronic infection is these animals may lead to the development of pancytopenia and potentially fatal hypoplastic bone marrow failure.

## *Ehrlichia*: Genome Characteristics and Intracellular Life Cycle

*Ehrlichia* species primarily infect macrophages and non-myeloid cells such as hepatocytes and endothelial cells. *Ehrlichia* exist in two forms within macrophages: (i) a small infectious nonreplicating dense core (0.4–0.6 μm), and (ii) a large, noninfectious reticulate form (0.4–0.6 μm × 0.7–1.9 μm) that undergoes binary fission within a cytoplasmic vacuole. The cytoplasmic vacuole contains *Ehrlichia morulae* (Latin for mulberry) which are visualized by Giemsa or Diff-Quick staining methods within infected monocytes or neutrophils from the peripheral blood smear.

Unlike other Gram-negative bacteria, *Ehrlichia* cell envelope lacks lipopolysaccharide (LPS) and peptidoglycan: two major PAMPS that are recognized by Toll-like receptors (TLRs) expressed by innate-immune and non-immune cells (12, 13). However, *Ehrlichia* cell outer membrane is enriched with proteins that express tandem repeat units (TRPs) (14–19). These TRPs are secreted into the target-cell cytosol via type I secretion system and are known to: (i) regulate host cell transcription factors involved in cell survival (20, 21); (ii) modulate cytoskeleton organization (21, 22); (iii) induce innate and adaptive immune responses; (23–27) and (iv) favor bacterial survival (28). *Ehrlichia* also express a 200 kDa protein resembling the host cell cytoskeletal protein, ankyrin, which is translocated to the host cell nucleus and binds to host cell chromatin (29, 30). Other outer membrane proteins include several members of the P28 family that mediate bacterial adhesion and invasion into macrophages (31, 32).

Internalization of *Ehrlichia chaffeensis* into macrophages is mediated by binding of the C terminus of an outer membrane entry-triggering protein (EtPE-C) to the mammalian glycosylphosphatidylinositol-anchored protein, DNase X, located on the host cell surface (33, 34). This binding triggers intracellular signaling via the transmembrane molecule, CD147, which recruits hnRNP-K to induce N-WASP activation (33, 35). Activation of N-WASP mediates actin polymerization and thus promotes bacterial internalization (36). Upon entry, *E. chaffeensis* is enclosed in endosome-like or phagosome-like compartments composed by lipid raft domains found in the host cell membrane that do not bind with lysosomes.

Mechanisms by which *Ehrlichia* spread from cell-to-cell at the early stages of infection, before host necrosis and/or pyroptosis are developed, are not completely understood. Studies have shown that *Ehrlichia* are transported through host cell filopodia during initial stages of infection but are released extracellularly during host cell necrosis at late stages of infection (37). Filopodia are cell membrane extensions that are organized by actin polymerization and continuous restructuring of filamentous actin (38). Inhibition of actin polymerization with cytochalasin D hindered the formation of filopodia and decreased bacterial burden (37). This indicated the requirement of actin polymerization for the spread and infection of new host cells during early infection (first 24 h). Transmission electron microscopy shows *Ehrlichia* cells migrating to new host cells within the confines of filopodia extensions without contact with the extracellular matrix (ECM) (3, 37). Thus, utilization of filopodia by *Ehrlichia* for cell-to-cell transmission may enable them to avoid exposure to the ECM, which is known to hinder bacterial nutrient acquisition, decrease bacterial replication, cause bacterial degradation and death via catabolic enzymes.

## Mouse Models for Ehrlichiosis

Initial models of HME were developed by infection of immunocompetent mice with *E. chaffeensis* (39) or infection of natural hosts such as dogs with *E. canis* (40). These studies defined several key parameters of the pathophysiology of the disease. However, *E. chaffeensis* is considered avirulent in immunocompetent mice as it causes an abortive infection that resolves approximately 10 days post-infection (39, 41). In addition, infection by *E. chaffeensis* in mice does not induce a measurable immune response or pathology that mimics ehrlichiosis in humans. In contrast, infection of immunocompromised mice with *E. chaffeensis* resulted in extensive tissue damage and persistent infection in different organs (liver, peritoneal cavity, brain, lung, and bone marrow) and mice became moribund within 24 days (42). Although utilization of immunocompromised mice has provided some information about the mechanisms of protective immunity and host resistance to ehrlichiosis, it did not address the mechanisms of host susceptibility to severe and potentially fatal ehrlichiosis. Therefore, other murine models of mild and fatal ehrlichiosis were developed by infection of immunocompetent C57BL/6 mice with mildly virulent *Ehrlichia muris* (*E. muris*) and highly virulent *Ehrlichia* species, *Ixodes ovatus* ticks (IOE), respectively (43–48). These murine models recapitulate the clinical outcome, the pathological aspects, and the laboratory findings in patients with HME.

*E. muris*-infected mice develop mild and self-limited disease with all animals surviving to infection. Mild disease in *E. muris*-infected mice is characterized by hepatosplenomegaly, elevated serum levels of liver enzymes, and minimal hepatic apoptosis (44). Although intraperitoneal infection with *E. muris* results in disseminated infection, ehrlichiae are cleared by day 10 post-infection (49). In contrast, the outcome of infection with IOE is dose- and route-dependent. Intraperitoneal (i.p.) infection of wild type (WT) C57BL/6 mice with high doses of IOE (10^3^–10^5^ organisms per mouse) causes severe and fatal disease characterized by primary liver dysfunction, marked by elevated liver enzymes and development of focal areas of hepatic apoptosis and necrosis, followed by excessive cytokine and chemokine production, referred to as “cytokine storm,” cell death and immunosuppression. Finally, these mice succumb to infection due to toxic shock-like syndrome with multi-organ failure on days 8–10 post-infection. On the other hand, intradermal infection of C57BL/6 mice with high doses of IOE (10^3^–10^5^ organisms per mouse) causes a mild disease similar to that induced by i.p. infection with *E. muris*.

Primary infection with *E. muris* also induces strong cell-mediated immune responses characterized by the development of protective CD4 Th1 cells and type I CD8 T cells. Additionally, infection promotes humoral immunity characterized by the generation of cross-reactive *Ehrlichia*-specific IgG antibodies that can recognize other *Ehrlichia* species (45, 50). Primary infection of WT C57BL/6 mice with *E. muris* in mice induces both cell-mediated and humoral memory immune responses that renders heterologous protection of WT mice against re-challenge with a lethal dose of IOE (44, 50, 51). On the other hand, primary infection of WT C57BL/6 mice with a sublethal dose of IOE, that causes mild disease, fails to provide homologous protection against re-challenge with a lethal dose of IOE (50).

## Cell-Mediated Immunity in Ehrlichiosis

**Figure 1:**
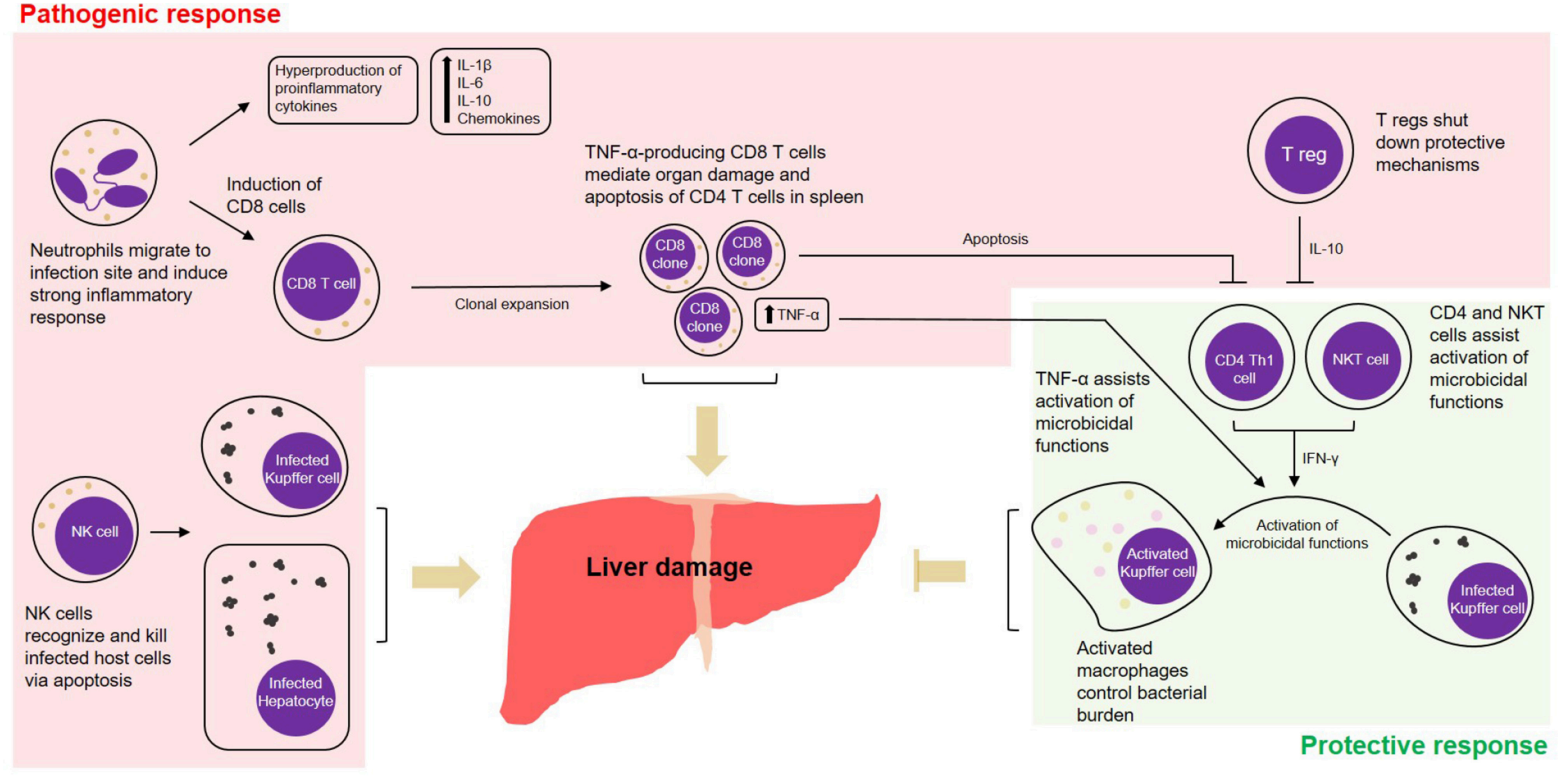
Cell-mediated pathogenic and protective responses in a model of fatal ehrlichiosis. Wild type C57BL/6 mice inoculated with virulent *Ixodes Ovatus Ehrlichia* (IOE) develop multi-organ failure and die between 8 and 10 days post-infection. Here we summarize protective and pathogenic mechanisms considering the cell-mediated immunity to *Ehrlichia* infection. Neutrophils migrate to infection site and induce strong pro-inflammatory response with hyperproduction of IL-1β, IL-6, IL-10, and chemokines. The role of neutrophils was also associated with induction of TNF-α-producing CD8 T cells that can either mediate organ damage or assist protective mechanisms. Another two important arms that mediate pathogenesis are the role of NK cells in eliminating infected cells in the target organs and the role of regulatory T cells (T regs), that end up shutting down protective mechanisms via IL-10. Protective mechanisms during infection by IOE include the induction of CD4 Th1 cells, that via IFN-γ activate on-site microbicidal functions of macrophages. NKT cells also play a role in stimulating such mechanism via IFN-γ.

Given that *Ehrlichia* is an obligate intracellular pathogen, cell-mediated immunity is thought to be key in protecting the host against infection. Despite this traditional line of thinking, it was seen that in ehrlichiosis many of the cell-mediated-immune mechanisms are more inclined toward deleterious effects. The outcome of infection is determined by the host's ability to balance between protective and pathogenic immune responses. For example, while CD4 Th1 and NKT cells seem to be protective, mechanisms mediated by CD8 T cells or NK cells can contribute to a pathogenic outcome. We found that liver injury and excessive cytokine and chemokine production/release followed by lethal *Ehrlichia* infection are mediated by several innate and adaptive immune cells, which are detailed below and summarized in Figure 1.

###  Neutrophils

Neutrophils serve as the frontline defenders against a variety of extracellular and intracellular pathogens including bacteria, fungi, and protozoa. Neutrophils are professional phagocytic cells that eliminate pathogens via phagocytosis and production of several antimicrobial peptides and molecules such as reactive oxygen species (ROS) (52). While neutrophils play a protective role during infections with other bacterial pathogens, our studies indicate that these cells are involved with a pathogenic outcome in ehrlichiosis. Depletion of neutrophils in IOE-infected mice enhanced protective immunity and resistance to fatal IOE infection (53). This was evidenced by attenuation of hepatic apoptosis and necrosis, two pivotal processes in the development of liver injury during infection with virulent IOE (53, 54). IOE-infected mice depleted of neutrophils showed reduced expansion of pathogenic CD8 T cells and their production of tumor necrosis factor-α (TNF-α). In this model, neutrophils were also associated with production of cytokines (IL-1β, IL-6, and IL-10) and several chemokines that are known to mediate migration of inflammatory and immune cells to sites of infection. The exact mechanism by which neutrophils promote the expansion of T cells is still unknown. However, as suggested by other studies, these cells could function as antigen presenting cells (APCs) to induce activation and clone expansion of T-cell subpopulations (55).

###  CD8 T Cells

Cytotoxic CD8 T cells are considered a major cell subset that confers protection against intracellular pathogens. These cells are capable of recognizing and eliminating infected cells via perforin/granzyme B activities, as well as via death receptors such as FAS and TNF-α receptor I/II (56, 57). In addition, production of TNF-α and IFN-γ by CD8 and CD4 T cells mediates activation of microbicidal functions in phagocytic cells. These cytokine-induced bactericidal mechanisms are marked by the production of nitric oxide by nitric oxide synthase 2 (NOS2), tryptophan degradation to kynurenine by indoleamine 2, 3-dioxygenase (IDO), and reactive oxygen species. CD8 T cells contribute to protective immunity during infection of mice with *E. muris*. This was evidenced by higher susceptibility of MHC class I deficient mice to infection. In this case, nearly 80% of the MHC class I deficient animals succumbed to infection while all WT control mice survive (58). Adoptive transfer of CD8 T cells and CD4 T cells from *E. muris*-infected mice into naive mice confers protection of recipient mice against IOE infection. Depletion of IFN-γ or TNF-α resulted in lethality of 75% of these animals (44, 59).

In contrast to mild *Ehrlichia* infection, virulent IOE induces expansion of TNF-α-producing cytotoxic CD8 T cells. Notably, β2 microglobulin knockout mice and TAP knockout mice, that are CD8 T cell deficient, are more resistant to lethal IOE infection compared to WT mice. This observation suggested that CD8 T cells play a role in immunopathology during fatal ehrlichiosis (44, 47). Further studies in knockout mice indicated that immunopathology mediated by CD8 T cells is likely driven by TNF-α overproduction (46). Mice that do not express TNF-α receptors I/II, after infection with IOE are marked by prolonged survival, attenuated host cell death and reduced liver injury. However, these animals present higher bacterial load when compared to similarly-infected WT mice, indicating that TNF-α produced by CD8 T cells plays a dual role mediating hepatic pathology and control of intracellular ehrlichiae.

###  NK Cells

Our studies indicate that NK cells contribute to liver damage during severe *Ehrlichia* infection via secretion of several pro- and anti-inflammatory cytokines and production of granzyme B/perforins that activate cell death mechanisms (49). Depletion of NK cells enhanced mice survival and decreased tissue damage in IOE-infected mice suggesting a pathogenic role of NK cells in fatal ehrlichiosis. Considering persistent infection, which is the case of animals that are inoculated with *E. muris*, NK cells play a protective role during primary and recall immune responses. Primary infection with *E. muris* promoted polarization of memory CD4 Th1 cells and memory-like NK cells in the spleen and liver at day 28 post-infection (60).

This memory/recall response protected *E. muris*-primed mice against re-challenge with *E. muris* or lethal dose of virulent IOE. Interestingly, depletion of NK cells in *E. muris*-primed mice decreased the numbers of memory CD4 T cells and antibody-producing B cells, and made animals more susceptible to infection after re-challenge with IOE (60). These findings suggest that memory-like NK cells generated following primary *E. muris* infection are key for the development of effective long-term immunity to fatal *Ehrlichia* infection.

###  CD4 Th1 and NKT Cells

Similarly to what occurs in other infections by intracellular bacteria, IFN-γ-producing CD4 T helper-1 (Th1) cells and natural killer T cells (NKT) mediate protective immunity against *Ehrlichia* (53, 61, 62). Production of IFN-γ by these cells activates microbicidal functions of macrophages enhancing bacterial clearance. However, these protective immune cells undergo apoptosis during late stages of severe *Ehrlichia* infection.

NKT cells are a subset of T cells that in addition to expressing T cell receptors, also express several molecules typically expressed by NK cells. NKT cells are CD1d-restricted cells that contribute to host defense against various microbial pathogens. A study by Mattner et al. highlighted an alternative pathway by which *Ehrlichia* activate NKT cells compared to other Gram-negative bacteria (61). This study showed that Gram-negative intracellular bacteria, such as *Salmonella*, trigger activation of NKT cells via recognition of endogenous lysosomal glycosphingolipid, iGb3, presented by CD1d molecules on dendritic cells. In contrast, antigen-specific activation of human and murine NKT cells against LPS-negative *E. muris* occurs following recognition of cell wall glycosylceramide-like molecules by CD1d molecules.

## Intracellular Innate Immune Surveillance in Ehrlichiosis

Innate immune receptors play a central role in immune surveillance by sensing pathogens and initiating protective immune responses. However, under certain conditions, the innate immune system plays a deleterious role by overreacting to pathogens and causing excessive inflammation, immunopathology, cell death, and tissue damage. Recent studies have uncovered the complexity of innate immune receptors that sense *Ehrlichia* species, and downstream fundamental eukaryotic pathways, such as inflammasome activation, autophagy, and type I IFN response, which have multiple immunological effects on infection and immunity. This review highlights the cellular and molecular mechanisms by which *Ehrlichia* infection is sensed by different innate immune receptors, as well as the mechanisms of crosstalk between autophagy and inflammatory signaling cascades. We also discuss how these events mediate both host resistance to infection and the pathogenesis of fatal ehrlichiosis.

###  Sensing of *Ehrlichia* by Pattern Recognition Receptors (PRRs)

We previously showed that fatal IOE infection differentially triggers upregulation of several PRRs in hepatic cells when compared to infection with *E. muris*. Liver tissues from IOE-infected mice express significantly higher levels of nucleotide-binding oligomerization domain-containing protein 2 (NOD2), a cytosolic PRR that senses microbial ligands such as peptidoglycan, and TLR2, a surface TLR that recognizes LPS and lipoproteins. Both NOD2 and TLR2 signal via MyD88 to induce activation of NF-κb and production of several cytokines and chemokines. We have shown that TLR2 deficient mice are more susceptible to severe and fatal IOE infection as indicated by higher mortality, increased bacterial burden, and presence of a significantly higher number of inflammatory foci and necrotic hepatocytes and macrophages 7 days after infection when compared to infected WT mice (54). In contrast, NOD2 deficient mice are more resistant to fatal ehrlichiosis when compared to infected WT controls (54). IOE-infected NOD2 deficient mice have less hepatic apoptosis and necrosis and were able to effectively clear ehrlichiae compared to infected WT controls. Enhanced resistance of NOD2 deficient mice to fatal ehrlichiosis was associated with restoration of T cells and NKT cell numbers, increased IFN-γ production, as well as decreased frequency of pathogenic CD8 T cells, suggesting that NOD2, at least in part, mediates disease progression following IOE infection.

We recently examined the role of MyD88 in the immune response to lethal IOE infection. We showed that MyD88-signaling functions under a “double-edged sword” manner, with MyD88 signaling playing protective and pathogenic roles in fatal ehrlichiosis by regulating two key innate immune events in macrophages: autophagy and inflammasome activation (63). As a host-protective mechanism, activation of MyD88 signaling by IOE attenuates bacterial survival and replication by inhibiting induction of autophagy, a host innate response that promotes survival and/or replication of *Ehrlichia* as suggested by studies from us and other investigators (63–66). Livers of IOE-infected MyD88 deficient mice or primary bone marrow derived macrophages (BMM) isolated from these animals and infected with IOE, show higher autophagy induction as characterized by autophagosome formation compared to similarly infected WT mice or WT-BMM. This effect was associated with increased bacterial burden *in vivo* or increased numbers of intracellular bacteria in *in-vitro* cultured BMM. Further, pharmacological enhancement of autophagy increased bacterial replication in IOE-infected WT-BMM compared to untreated and infected BMM. As a host-pathogenic mechanism, we found that MyD88 signaling blocks autophagy flux (i.e., autophagosome-lysosomal fusion) in macrophages following lethal IOE infection, which leads to inhibition of mitochondrial autophagy (i.e., mitophagy) as well as blockage of ehrlichial degradation via lysosome due to lack of colocalization of autophagosomes with lysosomes. Defective mitophagy and the blocking of autophagic flux in IOE-infected macrophages led to the accumulation of mitochondrial DAMPS (e.g., ROS and mitochondrial DNA) and PAMPs, which in turn resulted in activation of canonical and non-canonical inflammasome pathways. As discussed below, inflammasome activation plays a deleterious role in the pathogenesis of fatal ehrlichiosis as it promotes excessive and dysregulated inflammation, development of pathogenic innate and adaptive immune responses (mediated by NK cells, neutrophils, and CD8 T cells), and finally cell death and multi-organ dysfunction. This conclusion is supported by data showing that lack of MyD88-attenuated liver pathology, decreased the production of NF-κB-dependent pro-inflammatory cytokines (e.g., TNF-α) and inflammasome-dependent cytokines (IL-1α, IL-1β), and reduced the frequency of cytotoxic CD8 T cells. Attenuated immunopathology in MyD88-deficient mice was associated with enhanced survival and host resistance to fatal ehrlichiosis (63). Indeed, the lack of correlation between bacterial burden and survival in MyD88-deficient mice reinforces our previous conclusion using WT mice that severe and fatal ehrlichiosis is not due to an overwhelming infection, but rather due to immunopathology.

Further analysis of the upstream TLRs that signal via MyD88 revealed TLR9 as the major TLR that mediates MyD88 effector functions during fatal *Ehrlichia* infection. We also explored whether other extracellular and endosomal TLRs such as TLR2 and TLR7, respectively, are linked to MyD88 activation and subsequent inflammasome promotion in the liver. Both TLR7^−/−^ and TLR9^−/−^ mice produced lower amounts of IL-1β, with TLR9^−/−^ mice having significantly lower levels compared to WT and TLR7^−/−^ (63). Importantly, 85% of TLR9^−/−^ mice infected with lethal IOE survived until 60 days post-infection while all infected WT mice died between 10 and 12 days post-infection (63). These data support that the TLR9-MyD88 axis mediates host susceptibility and pathogenic responses during fatal ehrlichiosis. The results from the IOE model are consistent with observations from Rikihisa and coworkers using *E. chaffeensis* strain Wakulla, a virulent strain known to induce diffuse hepatitis in immunodeficient mice. *E. chaffeensis* infection induced TNF-α and IL-1β expression in the liver of immunodeficient mice and in isolated BMM (67). The expression of these cytokines was MyD88-dependent, but not dependent on TRIF, TLR2/4 or IL-1 receptor 1/IL-18 receptor 1. *In vitro* infection using human THP-1 cells (leukemia cell line) indicated that *E. chaffeensis* induces upregulation of IL-8, IL-1β, and TNF-α mRNAs as well as the extracellular regulated kinase 2 (ERK2) activation. Hence, *E. chaffeensis* Wakulla strain may induce inflammatory responses through MyD88-dependent NF-κB and ERK pathways, independent of TRIF and TLR2/4. Similarly, infection with *Anaplasma phagocytophilum*, another obligate intracellular bacterium that infects neutrophils and causes human anaplasmosis, triggers TLR2 signals which induce secretion of proinflammatory cytokines via NF-κB (68).

The role of MyD88 in host resistance and susceptibility to infections with other Rickettsial pathogens, such as *Rickettsia conorii* or *Rickettsia australis*; Spotted fever group (SFG) rickettsiae has also been examined. MyD88 deficient mice infected with either *R. conorri* or *R. australis* were more susceptible to infection than WT mice, suggesting a protective role of MyD88 in the immune response against SFG Rickettsiae (69, 70). Similar to its role in ehrlichiosis, MyD88 signaling mediated clearance of intracellular rickettsiae within macrophages and dendritic cells. Mechanistically, MyD88 signaling promoted IFN-γ production in mice, which is known to be critical for activation of microbicidal functions of macrophages. In addition, MyD88 signaling enhanced inflammasome activation, which was found to be a host-protective mechanism during *Rickettsia* infection.

###  Inflammasome Activation in Ehrlichiosis

Inflammasomes are high molecular weight protein complexes that consist of intracellular NOD-like receptors (NLRs), programmed cell death ASC (apoptosis-associated speck like protein) adaptor molecules and pro-caspases. Activation of inflammasomes leads to the activation of caspases which subsequently induce secretion of IL-1β and IL-18 proinflammatory cytokines (71–75). Inflammasome complexes include NLRP1, NLRP2, NLRP3, NLRP4, NRLP6, NRLP7, and NLRP12 (75). The canonical inflammasome activation pathway requires a priming event via triggering of a PRR, such as Toll-like receptors (TLRs), by a microbial or host ligand or by binding of a pro-inflammatory cytokine to its receptor, such as binding of TNF-α to TNF-α receptors (74–78). This ligand-receptor binding causes the MyD88-mediated activation of NF-κB leading to upregulation of pro-IL-1β, pro-IL-18, and several inflammasome complexes, such as NLRP3 (75). Next, assembly of the inflammasome complexes occurs upon binding to PAMPS or DAMPS (e.g., bacterial toxins, DNA, bacterial RNA and flagella, viral protein, host DNA and RNA, and host-derived ATP, glucose, cholesterol crystals, calcium pyrophosphate dihydrate, mitochondrial ROS and DNA, and amyloid β), which leads to activation of caspase 1, cleavage of IL-1β and IL-18, as well as pyroptosis. Recently, a non-canonical inflammasome pathway has been described in which cytosolic LPS activates the inflammasome via activation of caspase-11 which promotes caspase 1-dependent secretion of IL-1β and IL-18 as well as pyroptosis and release of IL-1α and high mobility group box 1 (HMGB1) (3, 73, 79–83). The inflammasome could function as a host protective mechanism to clear the infection and promote induction of protective adaptive immune responses against infectious agents, but it can also produce tissue injury, excessive inflammation, and immunopathology under dysregulated circumstances (75).

Employing murine models of ehrlichiosis, we have shown that LPS-negative IOE infection differentially induces upregulation of several inflammasome complexes including NLRP1, NLRP3, NLRC4, AIM2, and NLRP12 (54). Unlike infections with other intracellular bacteria, such as *Legionella* and *Mycobacterium tuberculosis*, inflammasome activation plays a deleterious role in the host response against *Ehrlichia*. This point is supported by several studies showing a strong link between the production of inflammasome-dependent cytokines, IL-18 and IL-1β, and the induction of pathogenic adaptive immune responses and liver damage. IL-18R knockout mice are more resistant to fatal ehrlichiosis than WT mice as marked by prolonged survival and decreased bacterial burden (48). A lack of IL-18/IL-18R signaling enhanced bacterial clearance, attenuated liver injury, decreased the production of pro-inflammatory cytokines (such as TNF-α), and decreased expansion of pathogenic TNF-producing CD8 T cells and NK cells following IOE infection. Further studies have shown that production of IL-1β is mediated by NLRP3, caspase 1, and caspase 11, indicating activation of both canonical and non-canonical inflammasome pathways (54, 63, 84). Notably, IOE-infected NRLP3-deficient mice effectively cleared *Ehrlichia*, but still displayed acute mortality and liver injury compared to IOE-infected WT mice. This suggests that pathogenic inflammasome activation during fatal IOE infection is only partially mediated by NLRP3 (84). The fact that virulent *Ehrlichia* infection causes caspase-11 production is eye-catching considering that the non-canonical inflammasome pathway is best-known to be activated by LPS directly binding to pro-caspase-11 in the cytosol, yet *Ehrlichia* species lack LPS as described above (85). Regardless, IOE-induced activation of caspase-1 and -11 production contributes to the development of pyronecrosis, liver injury, and excessive inflammatory responses (63).

###  Inflammasome Activation by Other Related Bacterial Species

The number of studies showing the involvement of other related bacterial species and inflammasome activation is minimal. In a study using mouse and human macrophages by Smalley and coworkers, it was shown that infection with *Rickettsia australis* activates NRLP3 inflammasomes in an ASC-dependent manner. ASC-inflammasome activation was characterized by significantly higher concentrations of IL-1β, IL-18, and mature caspase-1, however, BMM isolated from ASC^−/−^ mice displayed an attenuated inflammasome response with significantly reduced levels of pro-inflammatory cytokines and caspases (86). This work suggested that NLRP3 inflammasome contributes to cytosolic recognition of *R. australis*.

Similar to *Ehrlichia*, infection with *Anaplasma*, another tick-borne obligate intracellular bacteria that belongs to the family Anaplasmataecae (1, 2), triggers inflammasome activation (87). Even though sensing of *Anaplasma spp*. by PRRs remains mostly unidentified, it was seen that infection by *A phagocytophilum*, the etiologic agent of human granulocytic anaplasmosis, induces activation of the NLRC4 inflammasome. During *A. phagocytophilum* infection, cytosolic phospholipase A2 metabolizes arachidonic acid from phospholipids, which is converted to the eicosanoid prostaglandin E2 (PGE2) via cyclooxygenase 2 (COX2) and prostaglandin mPGES-1 activity. PGE2-EP3 receptor signaling culminates in the activation of the NLRC4 inflammasome and secretion of IL-1β and IL-18 (87).

###  Autophagy and Its Role in Pathogenesis

Degradation of cytoplasmic components is achieved through several pathways including macroautophagy, microautophagy and chaperone-mediated autophagy (88). Macroautophagy (standardly referred to as autophagy) is a highly conserved process by which cells recycle organelles and intracellular debris via degradation in the lysosomes. Autophagy is characterized by the regulated formation of double-membrane compartments known as phagophores. Phagophores encapsulate tagged intracellular materials, as well as intracellular pathogens for host defense purposes. Autophagic flux involves the maturation of phagophores into autophagosomes, which then fuse with lysosomes to form single-membrane autolysosomes, where degradation of autophagic cargo, recycling of proteins and ATP synthesis occur. Several autophagy-promoting molecules including, ATG5, ATG12, ATG16, ATG8, and Beclin-1, mediate the induction of autophagy (89–92). Any marked decrease in production of these proteins or in lipidation of ATG8/LC3 (LC3 is the mammalian homolog of yeast ATG8) attenuates the formation of autophagosomes and impairs the autophagy process overall. Autophagy has been implicated in many fundamental biological processes including aging, immunity, cell development and differentiation by regulating inflammation.

Autophagy vitally combats pathogens during most infection processes. However, several intracellular pathogens evade the host innate immune defense system by exploiting autophagy as a pathway to obtain nutrients, fatty acids, and carbohydrates required for intracellular survival and replication (93). Recent studies suggested that autophagy promotes survival and replication of obligate intracellular bacteria, such as *Ehrlichia chaffeensis* (64), *Anaplasma phagocytophilum* (94) and SFG Rickettsia (95). These bacteria would be examples of microorganisms capable of capturing nutrients through autophagy to promote bacterial growth and replication (17, 96–98). *E. chaffeensis* exploits autophagy proteins to obtain nutrients by secreting the protein ETF-1 (*Ehrlichia* translocated factor-1). ETF-1 targets the endosomal protein, RAB5, which is associated with an early endosome-like membrane-bound compartment that contains *Ehrlichia* but lacks bactericidal functions (i.e., ehrlichial inclusions). Binding of *E. chaffeensis* ETF-1 to RAB5 and autophagy-initiating class III phosphatidylinositol 3 kinase (PtdIns3K) is followed by Beclin-1, VPS34, and ATG5 recruitment to form an autophagosome that binds to the ehrlichial inclusion (64, 97, 99). Thus, *E. chaffeensis* hijacks the RAB5 autophagy pathway to create a non-microbicidal phagosomal complex to capture and deliver nutrients from already degraded cellular debris within the cytoplasm. In other words, the RAB5-associated phagosomal complex acts as an intracellular transport vesicle, in which captured cytosolic nutrients are delivered to *E. chaffeensis*. *E. chaffeensis* remains sheltered in endosome-like inclusions that were formed upon initial entry and internalization into the host cell (100).

Using a murine model of fatal ehrlichiosis, we have shown that IOE also exploits autophagy to survive and replicate within macrophages. Like *E. chaffeensis*, IOE induces autophagy in macrophages (the primary target cells) to survive and replicate within these phagocytic cells. Enhanced autophagy in IOE-infected MyD88^−/−^ BMM enhanced intracellular bacterial survival and replication (63). As a counteracting protective mechanism, signaling via MyD88 negatively regulates autophagy induction. Thus, MyD88 signaling during IOE infection plays a protective role by attenuating bacterial survival and replication via inhibition of autophagy induction (63). As mentioned above, MyD88 not only blocks autophagy induction, but also inhibits autophagosome-lysosomal fusion and, consequently, also inhibits the autophagic flux. Blocking autophagy induction with inhibitor compounds attenuated bacterial survival. The MyD88-mediated blocking of autophagic flux also resulted in defective elimination of mitochondrial DAMPs (i.e., defective mitophagy).

**Figure 2:**
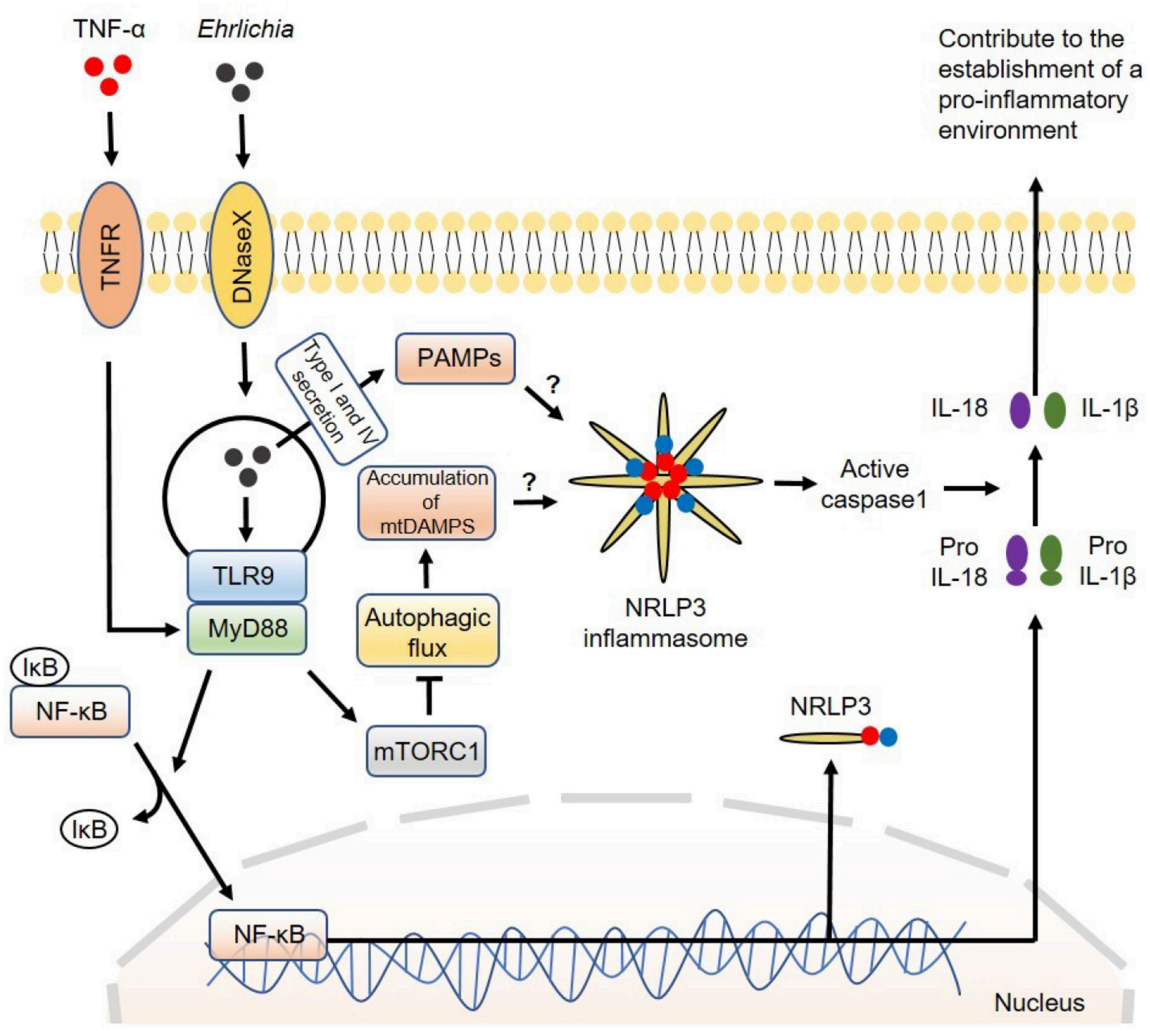
Model of canonical inflammasome activation involving regulation of autophagy induced by *Ehrlichia*. The canonical iflammasome activation pathway proposed in *Ehrlichia* infection involves two steps. In the first step, *Ehrlichia* invade the target cell and induce TLR9/MyD88 downstream targets to activate NF-κB. Activated NF-κB function as a transcription factor to upregulate NRLP3 complexes, pro-IL1β and pro-IL-18. In the second step, NRLP3 inflammasomes would be activated after recognition of Ehrlichial PAMPS which are secreted to the cytosol via type I and IV secretion systems. Accumulation of mtDAMPS originated by inhibition of autophagy via MyD88/mTORC1 signaling would also induce inflammasome activation. Inflammasome activation results in activation of caspase-1 that subsequently induces cleavage of pro-IL1β and pro-IL-18 into their mature forms. Mature IL-1β and IL-18 are then secreted to the extracellular space to contribute to the establishment of a pro-inflammatory environment. Direct arrow: positive regulation; blocking arrow: negative regulation; question mark (?) means hypothetical, and not experimentally proven.

Mechanistically, we found that MyD88 negatively regulates the autophagy process in IOE-infected macrophages via activation of mammalian target of rapamycin complex 1 (mTORC1), a member of the phosphoinositide 3-kinase (PI3K) family (63). The mTORC1 pathway is a well-known negative regulator of autophagy induction as it inhibits the binding of Beclin-1 to ULK: a vital first step in the formation of autophagosomes (101). It is not clear how MyD88 induces activation of mTORC1 in macrophages following infection with virulent IOE. The mTORC1 pathway is activated under homeostatic conditions because it promotes cell survival and proliferation. In contrast, mTORC1 is inhibited by amino acid starvation and cellular stress. Cell starvation activates mechanisms that cause upregulation of the autophagy process during times of critical metabolic need. It is possible that metabolic dysregulation during *Ehrlichia* infection may cause mTORC1 activation. Although mTORC1 mediates inhibition of autophagy, which works as a source of nutrients to *Ehrlichia*, it promotes host cell survival and masks *Ehrlichia* within the host cell. This paradoxical interaction between mTORC1 and autophagy is an important immune evasion mechanism that enables survival and replication of *Ehrlichia*. A model of the canonical inflammasome activation involving regulation of autophagy by the infection is described in Figure 2.

###  The Interplay Between Autophagy and Inflammasome During Infection With Other Rickettsial Pathogens

Similar to *Ehrlichia* infection and ehrlichiosis, autophagy appears to play a major role in the pathogenesis of other rickettsial diseases. Earlier studies in *Rickettsia* infection in mice suggested a role of autophagy in host defense against SFG *Rickettsia*. Endothelial cells infected with *Rickettsia conorii* displayed strong antirickettsial effect upon cytokine-induced nitric oxide (NO) stimulation. Ultrastructural analysis of infected endothelial cells revealed double membrane structures that appeared to be derived from the endoplasmic reticulum and contained rickettsiae. This has been described as phagolysosomes, which was postulated to be a process by which cytokine-activated endothelial cells eliminate intracellular *Rickettsia* (102). The anti-microbial role of autophagy in host response against *Rickettsia* was also suggested by a study comparing the growth kinetics of pathogenic and nonpathogenic rickettsiae in Vero and Hela cells. Cells infected with the non-pathogenic strain, *R. montanensis*, displayed the formation of autophagosomes, which was associated with a low number of intracellular organisms. On the other hand, superinfection of *R. montanensis*-infected cells with the pathogenic species, *R. japonica*, failed to control replication of both strains. This fact was associated with restriction or inhibition of autophagosome formation under superinfection conditions (103, 104). By employing computational biology techniques, Gong and coworkers showed that tissues or cells infected with *R. conorii* displayed the presence of tRNA-derived RNA fragments (tRFs) which may interact with transcripts associated with autophagy (105). Moreover, a recent study using ATG5^flox/flox^ mice suggested that autophagy promotes *Rickettsia australis* infection. Tissues or BMM from ATG5^flox/flox^ infected with *R. australis* harbor a higher number of *Rickettsia* compared to their counterparts of ATG5^flox/flox^ Lyz-Cre mice (in which macrophages only are deficient of ATG5, one of the autophagy genes that are essential for initiation of autophagosome). These data suggest that autophagy enhances rickettsial survival and/or replication. Notably, treatment of infected macrophages from ATG5^flox/flox^ mice with recombinant IL-1β attenuated rickettsial replication, indicating a protective function of IL-1β and inflammasome activation in the host response against *Rickettsia*. Importantly, these data suggest that autophagy induction following *R. australis* infection negatively regulates inflammasome activation and IL-1β production, a consistent conclusion with the occurrence of cross-talk between autophagy and inflammasome during *Ehrlichia* infection.

Similar to *Ehrlichia* and *Rickettsia*, autophagy induction and formation of autophagosomes support the survival and/or growth of *Anaplasma phagocytophilum*. This conclusion is supported by earlier studies by Galindo and colleagues showing that blood samples from pigs infected with *A. phagocytophilum* express higher levels of several genes including GJA1, integrin alpha-8, TSP-4, formin 1, Rho GTPase activating protein 5, keratin associated protein 26–1, calponin 3 and laminin receptor 1 (106). These genes are involved in cytoskeletal rearrangement and actin polymerization. Since autophagy requires cytoskeleton rearrangement for the formation of phagophore and internalization of invading microbes (107), this study suggested that infection with *A. phagocytophilum* induces modulation of autophagy. Elegant studies by Rikihisa and co-workers revealed the key mechanism by which *A. phagocytophilum* exploit autophagy to obtain nutrients for their survival. *A. phagocytophilum* were initially found to replicate in double-lipid bilayer membrane compartments that colocalize with LC3 and Beclin 1. Stimulation of autophagy by rapamycin favored *A. phagocytophilum* infection, and inhibition of the autophagosomal pathway impaired bacterial growth (94). Mechanistically, it was found that *A. phagocytophilum* induces autophagy through binding of *Anaplasma* translocated substrate 1 (Ats-1), type IV secretion system effector to Beclin 1-ATG14L pathway. This binding of the bacterial secreted molecule to the autophagosome enables *Anaplasma* to obtain nutrients and essential amino acids for their survival and replication (65, 66, 108).

###  Type I IFN Response in Ehlichiosis

#### Type I IFNs vs. Bacteria

Classical studies have focused on the role of type I interferons (IFN-I) in the induction of antiviral host defense (109, 110), however, others have demonstrated that IFN-I are also induced during infections with non-viral pathogens such as bacteria, mycobacterium, and parasites (87, 111–115). Despite this understanding, the mechanism of action of IFN-I in host defense against bacterial infections is still poorly elucidated. It looks like IFN-I exert disparate/dual roles depending on the type of bacterial infection. For example, infection with Gram-negative intracellular *Chlamydia trachomatis* can induce IFN-I, which restrict bacterial growth (116–118). Similarly, IFN-I are induced in mice infected with Gram-positive bacteria, such as *Group B streptococcus* and *Listeria monocytogenes*. In these cases, IFN-I are protective as evidenced by a higher susceptibility to infection in IFNAR^−/−^ mice compared to WT (119–122). On the other hand, IFN-I play a deleterious role during infection with other pathogens by promoting dysregulated inflammation and host cell death. For example, induction of IFN-I response in macrophages causes necroptosis upon infection with *Salmonella* (80). Additionally, IFN-I have also strong pro-inflammatory activities that contribute to high mortality rates in cases of septic shock (123). Factors that determine the protective or pathogenic function of IFN-I during infections with diverse pathogens are not entirely understood. However, studies suggested that the effector function of IFN-I depend on multiple bacterial and host factors including route and site of infection, bacterial virulence, and infectious dose. Table 1 summarizes the diverse contributions of IFN-I to host responses against several pathogens and the potential mechanism(s) by which they promote protective or deleterious roles.

**Table 1:** IFN-I response in bacterial infections.

**Bacteria**	**Mechanism of IFN-I induction and effects on host**	**References**
*Ehrlichia*	Trigger IFN-I responses through MyD88 and other unidentified receptor(s). IFN-I block autophagy and induce activation of the caspase-11-dependent non-canonical inflammasome pathway. By engaging caspase-11, *Ehrlichia* trigger pyroptosis in macrophages and induce assembly of the NLRP3 inflammasome or other yet unidentified inflammasomes that activate caspase-1 and IL-1β/IL-18 secretion.	(63)
*Brucella abortus*	IFN-I induction required STING- and MyD88- dependent signals.	(124)
*Coxiella burnetii*	Induction of IFN-I is mediated through TLR7/9, RIG-1 and NOD1 and NOD2, and signaling via MyD88 and IRF7.	(118, 125, 126)
*Salmonella enterica* serovar *Typhimurium*	Induction of IFN-I occurs by binding of LPS and nucleic acids to TLR4/TLR3 with signaling via TRIF pathway.	(127)
*Francisella tularensis*	IFN-I induce GBPs and activate AIM2 inflammasome leading to pyroptosis in macrophage and removal of replicative niche.	(128, 129)

We and others have shown that infection of WT mice with virulent *Ehrlichia* species (IOE) induces upregulation of IFN-I mRNA in liver tissues and secretion of IFN-I by plasmacytoid dendritic cells and macrophages in the spleen. IFNAR signaling plays a deleterious role in ehrlichiosis as evidenced by increased resistance of IFNAR^−/−^ mice to fatal IOE infection. IFN-I mediate host cell death and suppression of protective adaptive immune responses provided by CD4 Th1 cells. IFNAR signaling suppresses IFN-γ signaling, which impairs induction of antimicrobial pathways crucial for clearance of intracellular *Ehrlichia* (130, 131). Both murine IFNAR deficiency and neutralization of IFN-α and IFN-β individually decreased bacterial burden while correlating with increased IFN-γ production (130). Notably, increased IFN-γ in IOE-infected IFNAR^−/−^ mice was not essential for protection against fatal *Ehrlichia* infection (130). Studies from our laboratory suggest that attenuated inflammasome activation and enhanced autophagy are potential mechanisms that afford protection against fatal ehrlichiosis in IFNAR^−/−^ mice (54, 84). Deficiency of IFNAR resulted in attenuation of caspase-11 mediated-noncanonical inflammasome activation during IOE infection, also leading to reduced secretion of IL-1β and minimal cell death. IFN-I signaling also contributed to a pathogenic immune response during fatal *Ehrlichia* infection due to induction of cytotoxic TNF-α-producing pathogenic NK cells, neutrophils, and CD8 T cells that cause tissue damage. Additionally, IFNAR signaling induced pyroptosis or pyronecrosis in ehrlichiosis, which is consistent with other infection models. Expression of IFNAR on non-hematopoietic, but not on hematopoietic cells, is likely to be an important point of modulation of immunopathology during fatal *Ehrlichia* infection. Based on the above studies, we propose that endothelial cells and/or hepatocytes are major cellular sources of deleterious IFNAR signaling for the following reasons: (i) although the primary target cells are macrophages, *Ehrlichia* species infect other parenchymal cells, such as hepatocytes and endothelial cells, as shown by immunohistochemistry staining of infected liver tissues; (ii) fatal ehrlichiosis in humans and mice are associated with hepatic apoptosis and necrosis which correlate with IFN-I response in mice suggesting that hepatocytes play a role in the pathogenesis of the disease; and (iii) *Ehrlichia* exhibit tropism for microvascular endothelium leading to vascular inflammation and dysfunction/damage; this represents one of the key features of Ehrlichial pathogenesis, especially in patients with meningitis or encephalitis.

#### Regulation of Inflammasome Activation by Type I IFNs

**Figure 3:**
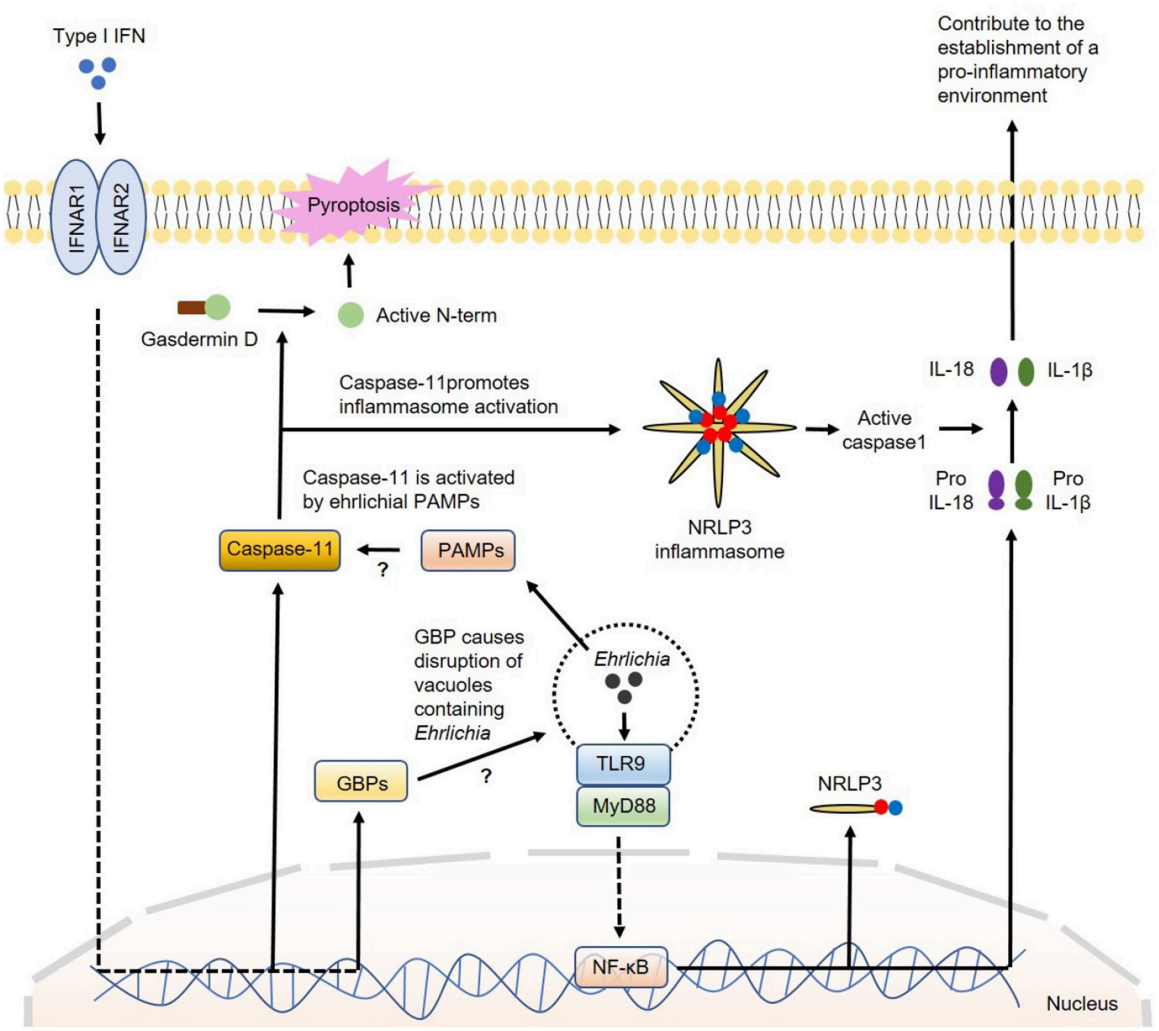
Model of regulation of inflammasome activation by type I IFNs in *Ehrlichia* infection. During *Ehrlichia* infection, type I IFNs are thought to regulate inflammasome activation under a non-canonical fashion. Initially, *Ehrlichia* invade the target cell and induce TLR9/MyD88 signaling to upregulate NRLP3 complexes, pro-IL1β and pro-IL-18 via NF-κB. Type I IFNs from autocrine or paracrine sources would signal through IFNAR to upregulate GBPs and caspase-11. GBPs would disrupt vesicles containing *Ehrlichia* allowing the escape of ehrlichial PAMPs to the cytosol. Ehrlichial PAMPs would bind caspase-11 and subsequently induce inflammasome activation followed by secretion of mature IL-1β and IL-18 (as in the canonical pathway) and cleavage of gasdermin D resulting in pyroptosis. Direct arrow: positive regulation; blocking arrow: negative regulation; question mark (?) means hypothetical, and not experimentally proven.

How IFN-I regulate canonical or non-canonical inflammasome activation is not completely understood. The major inflammasome complex that is regulated by IFN-β is NLRP3. Several studies showed that TLR4-TRIF axis regulates caspase-11 expression and non-canonical NLRP3 inflammasome-mediated host defense against enteropathogens, such as *Escherichia coli, Citrobacter rodentium* and *Salmonella Typhimurium* (132). Further studies indicated that IRF3 and IFNAR signaling during infections with intracellular bacterial pathogens that reside within phagosomes are required for caspase-11 expression and activation of the NLRP3 inflammasome and pyroptosis (80, 133). Recent studies have demonstrated that IFN-I promote caspase-11 activation by inducing several genes which are members of the IRG and GBP families of IFN-inducible GTPases. These GBP proteins are found to be essential for the activation of caspase-11–dependent pyroptosis in response to infections with *Legionella pneumophila* as they induce disruption of vacuoles containing *Legionella* causing the release of bacterial LPS into the cytosol (72, 117, 134–136). Cytosolic LPS is a known PAMP that triggers activation of caspase-11. Other studies have shown that IFN-I-induced GBP protein expression is required for the full induction of pyroptosis by LPS delivered to the cytoplasm independent of infection. Pyroptosis has been widely associated with cleavage of gasdermin D with release of its N-terminal portion that can destabilize the membrane creating pores leading to cell death (137). Collectively, these studies support that GBP proteins play a role in the detection of cytoplasmic LPS and/or the subsequent activation of the noncanonical inflammasome leading to pyroptosis (116, 117). Interestingly, while the induction of pyroptosis by cytoplasmic *L. pneumophila* LPS appears to be strictly dependent on GBP proteins, cytoplasmic LPS derived from Enterobacteriaceae can trigger pyroptosis in the absence of GBP proteins, however, with diminished efficiency (117, 124). This discrepancy could be due to the difference in the structures of LPS and TLR ligands among these bacterial species. Nevertheless, whether IFN-I-induced GBP proteins contribute to activation of caspase-11 by *Ehrlichia* that reside within vacuole or phagosome remains elusive. A model for the participation of IFN-I in the regulation of inflammasome activation in *Ehrlichia* infection is proposed in Figure 3.

## Conclusion

The last decades were marked by several advances in the description of how *Ehrlichia*-host interactions are established. Animal models have provided valuable information regarding the cell-mediated mechanisms that govern protection and immuomopathogenesis. Even though there is no vaccination available against *Ehrlichia*, understanding the mechanisms that protect the host against infection will assist us in the development of an effective vaccine. In terms of cell-mediated immunity, we currently know the importance of CD4 Th1 and NKT cell responses in protecting the host. Therefore, vaccine prototypes that prioritize such responses would represent promising tools within the vaccine development pipeline. Regarding the intracellular innate immune mechanisms and their nuances in modulating *Ehrlichia* infection, targeting autophagy and inflammasome activation can also represent promising options in drug development. By inhibiting autophagy and attenuating inflammasome activation via modulation of MyD88 and IFN-I signaling it would be possible to control the disease, once these mechanisms were categorically highlighted as key in *Ehrlichia* pathogenesis. Despite the current understanding, several gaps in knowledge concerning *Ehrlichia*-host interaction are yet to be solved for better planning of translational strategies against the disease.

## Author Contributions

TT wrote the original draft of the manuscript. EO created the figures, captions, and wrote part of the manuscript. SH reviewed the literature, draw the table and wrote a section of the review. JW contributed to a section of the review. BG wrote a section of the review and edited section written by TT. TT, EO, SH, AE, JW, BG, and NI reviewed and edited the manuscript.

### Conflict of Interest Statement

The authors declare that the research was conducted in the absence of any commercial or financial relationships that could be construed as a potential conflict of interest.
